# STUDY DESIGN OF THE AGING AND COGNITIVE HEALTH EVALUATION IN ELDERS (ACHIEVE) TRIAL

**DOI:** 10.1093/geroni/igae098.0609

**Published:** 2024-12-31

**Authors:** Alison Huang

**Affiliations:** Johns Hopkins Bloomberg School of Public Health, Baltimore, Maryland, United States

## Abstract

This presentation describes the design, interventions, participants, and primary and exploratory outcomes of the Aging and Cognitive Health Evaluation in Elders (ACHIEVE) study. The ACHIEVE study is a multicenter, randomized controlled trial designed to test the effect of a best-practice hearing intervention vs. health education control on cognitive decline (primary outcome) and other exploratory health outcomes, such as physical activity and function, over 3 years. Participants were 977 community-dwelling adults aged 70-84 years with untreated hearing loss and without substantial cognitive impairment. The ACHIEVE study was partially nested within the Atherosclerosis Risk in Communities [ARIC] study; participants were recruited from the ARIC study (n=238) and de novo (n=739) from the surrounding communities of four U.S. sites. Participants were randomized 1:1 to hearing intervention or health education control and followed for 3 years. The study’s primary outcome was cognitive decline; measures of physical activity (accelerometry-based and self-reported physical activity), fatigue, and physical function were collected as exploratory outcomes. Participants were median age 76.4 years, 53.5% female, 87.8% White, and 53.3% held a Bachelor’s degree or higher. Compared to the ARIC cohort, participants recruited de novo were younger, had fewer chronic conditions, higher cognitive function, and worse self-reported hearing-related quality of life at baseline. Differences in participant characteristics by recruitment source are important considerations in the interpretation of intervention effect. This presentation provides important background for contextualization of ACHIEVE study findings on physical activity and function presented in this symposium.

